# Dysfunction of Self-Control in Facebook Addiction: Impulsivity Is the Key

**DOI:** 10.1007/s11126-019-09683-8

**Published:** 2019-11-26

**Authors:** Andrzej Cudo, Małgorzata Torój, Marcin Demczuk, Piotr Francuz

**Affiliations:** 1 Department of Experimental Psychology, The John Paul II Catholic University of Lublin, Aleje Racławickie 14, 20-950 Lublin, Poland; 2 Department of Emotion and Motivation Psychology, The John Paul II Catholic University of Lublin, Aleje Racławickie 14, 20-950 Lublin, Poland; 3 Specialist Clinic for Psychoprophylaxis and Family Therapy in Lublin, Żołnierzy Niepodległej 1, 20-078 Lublin, Poland

**Keywords:** Facebook addiction, Self-control, State orientation, Impulsivity

## Abstract

Facebook is one of the most popular social network sites and communication platforms. However, besides many positive elements related to the use of this network site, in some cases it may lead to addiction. Therefore, the main aim of our study was to identify Facebook addiction predictors, in particular, to verify whether impulsivity, as a dimension of self-control, is an important predictor of this type of addiction. We also examined whether Facebook addiction predictors such as time spent using Facebook, use of Facebook smartphone apps, state orientation and female gender would be significant in our model of Facebook addiction. The 234 participants in the study were assessed using the Facebook Intrusion Questionnaire, the Brief Self-Control Scale and the Action Control Scale. Impulsivity as a dimension of self-control, action control, amount of time spent on Facebook, Facebook app use and gender were found to be related to Facebook addiction. Specifically, a high level of impulsivity, more time spent using Facebook, female gender and Facebook smartphone app use are predictors of Facebook addiction. However, the relation between state orientation, restraint as a dimension of self-control and Facebook addiction was insignificant. Our results may indicate the role of impulsivity as a dimension of self-control in Facebook addiction. In addition, they may suggest that self-control should be taken into account not only as a one-dimensional but also as a multidimensional construct in Facebook addiction research. Our findings may also contribute to the better preparation of prevention and therapeutic programmes for people at risk of Facebook addiction.

## Introduction

Social network sites (SNSs) are the platforms where people may exchange their information, share experiences and get to know new people. There are many positive influences of SNSs on people’s social lives [1]. People who have problems building relationships with new people may start doing this via the Internet and feel part of a group or they may take part in webinars. There are some reports that also underline a positive function of social media particularly for psychiatric patients (positive interaction and social improvement) [2] and for children (who may find support groups) [3]. The use of SNSs nowadays is quite high and there are a growing number of research articles that describe their negative impact on users [4].

One of the most popular SNSs is Facebook [4], thus interest in Facebook is growing among researchers because, apart from its positive function, it may cause specific problems that have an impact on the health and well-being of its users [3, 5, 6]. Some research undermines an obvious correlation between Facebook use and mental problems such as somatization, depression or anxiety [7]. With this in mind, it is worth differentiating if we should consider Facebook use and time spent using it, or whether we should be more focused on Facebook addiction or Facebook intrusion. The latter is used to underline the phenomenon of overusing this social network and its similarity to other addiction mechanisms.

Facebook addiction is defined as “excessive involvement in Facebook activities and is a frequent cause of problems in everyday social functioning” [8]. This is close to the definition of an SNS addiction proposed by Andreassen, Torsheim and Pallesen [9]: “being overly concerned about SNSs, to be driven by a strong motivation to log on to or use SNSs, and devote so much time and effort to SNSs that it impairs other social activities, studies/job, interpersonal relationships, and/or psychological health and well-being”. Both definitions underline the impairment of social functioning. Many people start to spend more time in virtual relationships than in direct contact. However, one of the most important signs of overusing Facebook is that people stop controlling the time spent on scrolling. Therefore, it seems very important to find out what the basic control mechanisms are that make people, who try to use Facebook in a positive social way, start to be addicted. The main aim of our research was to find out what the predictors of Facebook addiction are.

According to Turel and Qahri-Sarem [10], one of the mechanisms important for this type of addiction is a problem with cognitive and behavioural control. They measure behavioural and mental control treated mostly as the ability to stop the problematic use of Facebook and stop thinking about it. In our research, we try to measure control as the ability to self-control, defined as the capacity to alter one’s responses. These responses can be consciously altered to bring them in line with standards such as ideals, values, morals and social expectations, and to support the pursuit of long-term goals [11]. Self-control is an important predictor of life success and health [12]. In this context, a low level of self-control is characterized by behaviours including impulsiveness, risk-taking, short time perspective in thinking and addictions such as substance, game, Internet, gambling and Facebook addiction [12–16]. Also, it has been shown that the deficit in self-control is linked to the increased use of SNSs at work [9]. However, Carver [17] indicates that self-control consists of two dimensions: restraint and impulsivity. Restraint is characterized by a tendency to be deliberative or disciplined and engage in effortful control, whereas impulsivity is characterized by a tendency to act spontaneously and without deliberation. These dimensions are negatively related, function simultaneously and compete with each other for impact on behavioural outcomes [17, 18]. However, previous studies have shown that impulsivity is a predictor of drug addiction [19], alcohol dependence, gambling disorder [20], Internet addiction [21, 22] and SNS addiction [23], therefore it is possible that a high level of impulsivity can play an important role in Facebook addiction. Hence, it can be assumed that a high level of restraint should be a factor that protects against Facebook addiction.

As with previous research [16], we also considered action–state orientation, which is connected with the volitional processes that people use to engage in goal-directed behaviours. Kuhl [24] noticed that people who act under high-demand circumstances differ in ego depletion on subsequent tasks, referring to their orientation toward action or state preservation. According to his research, people who are action oriented are more effective in self-motivation, are more decisive and proactive, whereas people who are state oriented are more passive because of their hesitation and preoccupation [25]. This may mean that if a Facebook stimulus appears it may be more difficult for people who are state oriented to be resistant to it. In our research, we assumed that people who are more state oriented might be more vulnerable to Facebook addiction.

We also consider the time spent using Facebook and Facebook smartphone app use as two of the main predictors. Cudo et al. [26] confirmed that these variables are important for Internet addiction so it may be concluded that it is also an important predictor of Facebook addiction. There is also some evidence that gender may be one of the predictors of Facebook addiction. In the context of high SNS use, addiction had a stronger negative association with female than male well-being. Some research shows that female gender is a predictor of Facebook addiction [27, 28] but there is no clear evidence for this. Some of the researchers who tested the role of gender show that there are no correlations between SNSs and gender [9], or between Facebook and gender [10]. However, if there is no clear evidence it is worth trying to verify if gender is one of the predictors for Facebook addiction.

In conclusion, the main aim of this study was to explore how dimensions of self-control, with particular emphasis on impulsivity, and action–state orientation are related to Facebook addiction. Another aim of the study was to examine the weekly number of hours spent using Facebook, Facebook smartphone apps and female gender as predictors of this type of addiction. Taking into account the theoretical considerations and previous findings, we state the following hypotheses:

- H1: Impulsivity will be positively related to Facebook addiction.
- H2: Restraint will be negatively related to Facebook addiction.
- H3: State orientation will be positively related to Facebook addiction.
- H4: Weekly number of hours spent using Facebook and Facebook smartphone app use will be positively related to Facebook addiction.
- H5: Female gender will be a predictor of Facebook addiction.

## Method

### Participants and Procedure

The sample consisted of 382 individuals who completed the paper questionnaire. However, video game players were excluded from the study because previous research has shown that their cognitive functioning may differ from that of non-players [29]. Therefore, data from 234 participants took part in the analysis (91.5% women). The mean age of the participants was 24.86 years (SD = 6.42; range = 18–54). All participants were volunteers and received no monetary reward. They were informed that their responses would be anonymous and the study was conducted in compliance with the Declaration of Helsinki.

### Instruments

1. The Facebook Intrusion Questionnaire [8] was used, measuring eight aspects of Facebook addiction (cognitive salience, behavioural salience, interpersonal conflict, conflict with other activities, euphoria, loss of control, withdrawal, relapse and reinstatement). The scale includes eight items to which answers are given on a seven-point scale from 1 (*strongly disagree*) to 4 (*strongly agree*): Cronbach’s α = 0.77. Example items are: “I lose track of how much I am using Facebook” and “I have been unable to reduce my Facebook use”.
2. The Brief Self-Control Scale [12] adapted into Polish by Pilarska and Baumeister [30] was used to measure self-control. The scale includes 13 items to which answers are given on a five-point scale from 1 (*not at all like me*) to 4 (*very much like me*): Cronbach’s α = 0.84. Example items are: “People would say that I have iron self-discipline” and “I often act without thinking through all the alternatives”. In line with previous studies, the scale was divided into two subscales: restraint (Cronbach’s α = 0.72) and impulsivity (Cronbach’s α = 0.73) [18, 31].
3. The Action Control Scale (ACS-90) [32] adapted into Polish by Marszał-Wiśniewska [33] was used to measure action control. The scale includes 36 items to which answers are given by choosing one of two possible answers (e.g. “When I’m stuck in traffic and miss an important appointment”: (A) *At first, it’s difficult for me to start doing anything else at all*; (B) *I quickly forget about it and focus on something else*). The scale is divided into three subscales: AOF: action orientation subsequent to failure vs. preoccupation (Cronbach’s α = 0.74); AOD: prospective and decision-related action orientation vs. hesitation (Cronbach’s α = 0.76); AOP: action orientation during (successful) performance of activities (intrinsic orientation) vs. volatility (Cronbach’s α = 0.55).
4. Short questionnaire related to demographics information as well as gender, age, weekly number of hours spent using Facebook and Facebook smartphone app use.

## Results

The descriptive statistics – means (M) and standard deviations (SD) of all the variables and Spearman’s rho correlations between the variables – are presented in Table 1. Facebook addiction correlated positively with the weekly number of hours spent using Facebook, Facebook smartphone app use and impulsivity. Also, the negative relationship between Facebook addiction and AOD was significant. Moreover, a negative correlation between AOF, AOD and number of hours per week spent using Facebook was demonstrated.

**Table 1:** Means, standard deviations and correlations between variables

Variables	M	SD	(1)	(2)	(3)	(4)	(5)	(6)	(7)	(8)
(1) Facebook addiction	19.86	8.95								
(2) Facebook use (hours)	19.71	19.53	0.47***							
(3) Facebook app use	0.90	0.30	0.32***	0.11						
(4) Gender	0.09	0.28	−0.08	−0.06	−0.05					
(5) Self-control: restraint	11.91	2.49	−0.15*	−0.03	−0.13*	0.02				
(6) Self-control: impulsivity	10.33	2.79	0.27***	0.08	0.06	0.04	−0.26***			
(7) AOF	4.49	2.74	−0.09	−0.13*	−0.01	0.10	0.08	0.07		
(8) AOD	6.20	2.91	−0.17*	−0.14*	−0.11	0.05	0.33***	−0.26***	0.47***	
(9) AOP	8.02	2.29	0.09	0.06	0.03	0.03	−0.04	−0.24***	−0.08	0.09

Gender: 0, women; 1, men

AOF, action orientation subsequent to failure vs. preoccupation; AOD, prospective and decision-related action orientation vs. hesitation; AOP, action orientation during (successful) performance of activities vs. volatility

*p < 0.05; **p < 0.01; ***p < 0.001

In order to estimate the regression model for Facebook addiction, analysis of structural equations based on an asymptotically distribution-free method was used. The χ^2^, χ^2^/df, RMSEA (root-mean-square error of approximation), SRMR (standardized root-mean-square residual), GFI (goodness-of-fit index), CFI (confirmatory fit index), IFI (incremental fit index), TLI (Tucker–Lewis index) and NFI (normed fit index) statistics were applied as measures of the model fit [34, 35]. The proposed model will fit the dataset well when the value of χ^2^ is statistically insignificant (*p* > 0.05) and the value of χ^2^/df is lower than 2. Values of RMSEA lower than 0.05 and values of SRMR lower than 0.08 may suggest that the model is a good fit to data. Also, the model will fit well to a dataset when values of GFI, CFI, IFI, TLI and NFI are higher than 0.95 [34, 35]. The analysis takes into account the relationship between the self-control and action control dimensions.

**Fig. 1:**
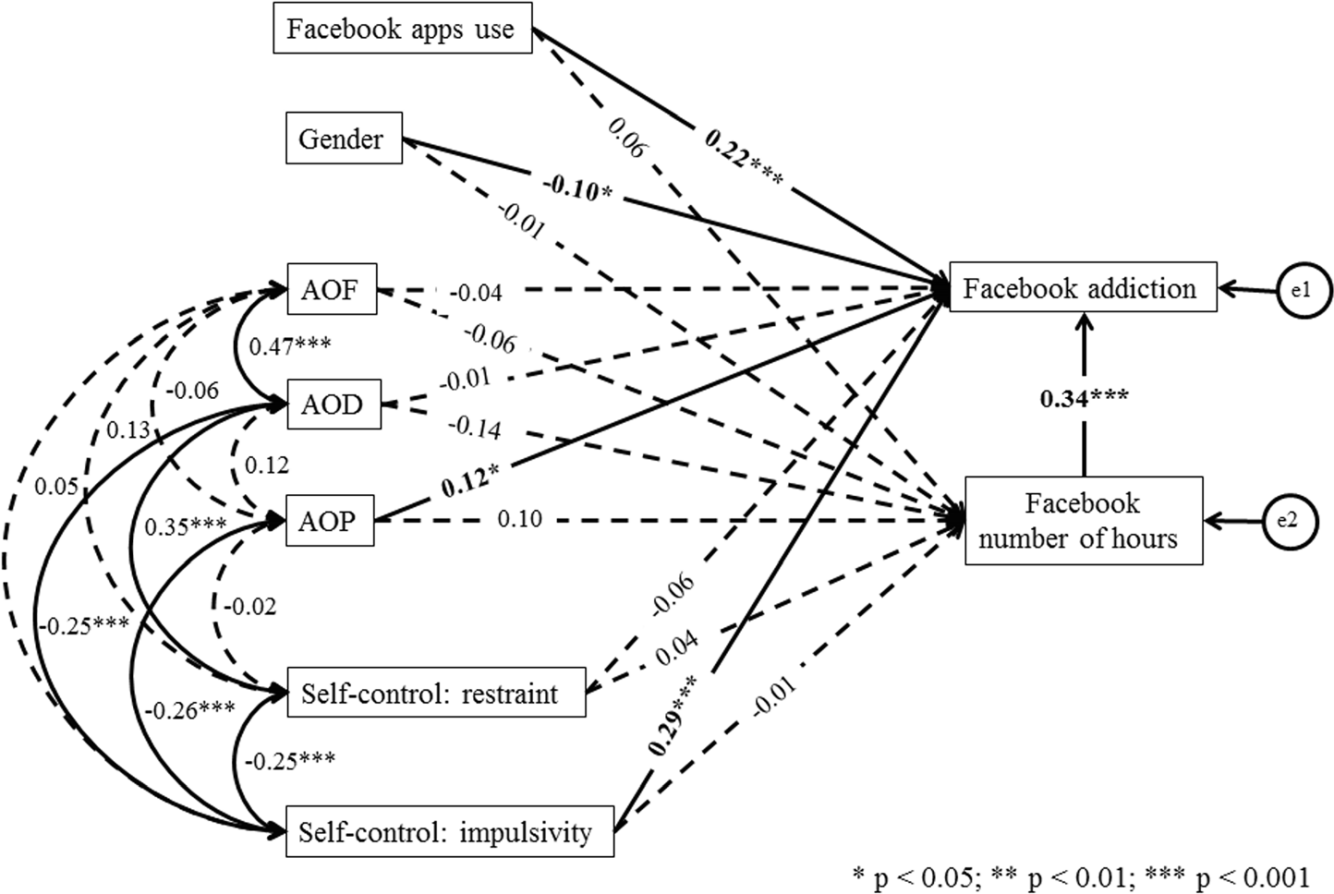
Structural model depicting predictors of Facebook addiction

The analyses carried out indicate that the regression model for Facebook addiction fits well: χ^2^_(df = 11)_ = 12.28; *p* = 0.343; χ^2^/df = 1.12; RMSEA = 0.022; SRMR = 0.040; GFI = 0.992; CFI = 0.994; IFI = 0.995; TLI = 0.982; NFI = 0.954. The proportion of variance in Facebook addiction accounted for by the set of independent variables in the model is 0.29. The amount of time spent on Facebook (β = 0.34; *p* < 0.001), Facebook smartphone app use (β = 0.22; *p* < 0.001), AOP (β = 0.12; *p* = 0.030) and impulsivity (β = 0.29; *p* < 0.001) were positively and significantly related to Facebook addiction. Also, a significant path was confirmed for the relation of gender (β = −0.10; *p* = 0.015) with Facebook addiction. Other paths between predictors and Facebook addiction were statistically insignificant. Also, none of the paths between the predictors and the weekly number of hours spent using Facebook were found. The findings also showed a significant correlation between impulsivity, restraint and AOD. There was a significantly positive correlation between AOD and AOF. Also, the negative correlation between impulsivity and AOP was significant. All other correlations were statistically insignificant (see Fig. 1).

## Discussion

The main aim of our study was to identify Facebook addiction predictors, in particular, to verify whether impulsivity, as a dimension of self-control, is an important predictor of this type of addiction. We also examined whether the Facebook addiction predictors shown in previous studies [5, 16, 27, 28, 36], such as number of hours spent using Facebook, use of Facebook smartphone apps, state orientation and female gender, would be significant in our model of Facebook addiction. We also checked whether the variables analysed in our study would be predictors of Facebook usage time. The findings indicate that impulsivity, action control, amount of time spent on Facebook, Facebook app use and gender are related to Facebook addiction. As we hypothesized, a high level of impulsivity, more time spent using Facebook, female gender and Facebook smartphone app use are predictors of Facebook addiction. However, we cannot confirm the relation between state orientation, restraint and Facebook addiction.

As we hypothesized, our results showed the relation between impulsivity (as a self-control dimension) and Facebook addiction. Specifically, a high level of impulsivity was an important predictor for this type of addiction. Our findings are in line with previous research [23], which indicated that impulsivity is one of the predictors of addiction to SNSs. Also, Gerson et al. [37] showed that participants who had high impulsivity used Facebook more intensively than those with low impulsivity. Moreover, it has been shown that impulsivity is also a predictor of other addictions, such as substance addiction, gambling, game addiction and Internet addiction [14, 19–22]. Our results may indicate that impulsivity, characterized as lack of planning or forethought and lack of consideration of the consequences [17], is a significant predictor of Facebook addiction and people who have a high level of impulsivity may have problems with dysfunctional Facebook use. Moreover, considering that impulsivity may also be a factor in procrastination [38, 39], it should be noted that procrastination has been shown to be a predictor of Facebook addiction [40]. Taking all this into account, it can be assumed that impulsiveness may play a crucial role in this type of addiction, not only directly but also indirectly.

Contrary to our expectations, we showed no relation between restraint and addiction in our model. One of the possible explanations may be related to spontaneous hedonic reactions to Facebook stimuli [41]. Van Koningsbruggen et al. [41] showed that the frequency of Facebook use was positively associated with spontaneous hedonic reactions to Facebook stimuli and that such reactions were related to self-reported craving to use Facebook. In this context, it should be noted that restraint, unlike impulsivity, is characterized by the tendency to reflect and deliberate before acting and it has limited effect on the perceived hedonistic tone related to action or stimulus [17, 18]. On the other hand, people with a high level of impulsivity may react spontaneously without reflecting on the consequences when vital (for them) stimuli such as Facebook occur. In summary, a restraint – characterized as the tendency to deliberate an action before it is carried out – may have less relevance than impulsivity – characterized as the tendency to act spontaneously in response to an important (for them) stimulus – for Facebook addiction. This assumption may be supported by the results of other research based on Dual System Theory (also known as reflective–impulsive theory of the mind) [42], which showed that SNS addiction is primarily related to dysfunction of the impulsive system rather than the reflective system [10, 43, 44]. The impulsive system is responsible for generating impulsive behaviour, whereas the reflective system serves regulatory goals and is responsible for higher-order mental operations [42]. In this context, the results of our research are likely to be consistent with the above studies [10, 43, 44] and indicate that restraint, defined as the tendency to be deliberative or disciplined and engage in effortful control, is not related to Facebook addiction. On this basis, it can be assumed that a spontaneous reaction to Facebook-related stimuli and thoughts may be important for this type of addiction. People with high impulsivity may react spontaneously to these thoughts and stimuli and consequently spend more time on Facebook, which entails a higher risk of becoming addicted. In this situation, restraint may be of little importance in responding to the Facebook-related stimulus and thoughts, and consequently to addiction.

We hypothesized that state orientation would be positively related to Facebook addiction. However, our results do not fully show such a relation and we only demonstrated a significant relation between performance-related action orientation (AOP) and Facebook addiction. It should be noted that failure-related (AOF) and decision-related (AOD) action orientation subscales measure a single construct that is associated with the ability to detach from thoughts about unpleasant past experience and assess difficulties in initiating an intended action [32]. The performance-related action orientation (AOP) subscale also measures a second construct associated with the ability to stay focused on self-initiated and pleasant activities [32], therefore our results may suggest that individuals with a higher level of this type of ability may have greater problems with dysfunctional Facebook use. Taking into account that impulsivity may be related to reactivity to Facebook-related stimuli, it may be assumed that the ability to stay focused on self-initiated and pleasant activities may contribute to maintaining a pleasant activity related to using this SNS, hence our results may point to a mechanism for starting and maintaining Facebook usage that may lead to addiction. It should be noted that a study by Błachnio and Przepiórka [16] demonstrated that failure-related action orientation (AOF) was a predictor of Facebook addiction, but in contrast to our research they did not take into account the relationship between self-control and action control dimensions. Further research is therefore needed to describe better the mechanism of Facebook addiction related to self-control and action control.

As we hypothesized, our results demonstrated a relation between the number of hours spent using Facebook, Facebook smartphone app use and Facebook addiction. More hours spent using Facebook and Facebook smartphone apps led to higher levels of this type of addiction. These results are in line with previous studies, which indicated a positive correlation of Internet addiction to the frequency of Facebook use, number of friends and time spent using this SNS [26, 36]. Other studies also showed a link between the use of mobile SNS apps and SNS addiction [45, 46]. One possible explanation for the relationship between these predictors and Facebook addiction may be linked to a change in social contact. Individuals may transfer social relations from real life to virtual life as they increase the time using Facebook [47]. This may be especially possible when individuals use Facebook smartphone apps and, as a consequence, they may feel in constant contact with others and have less fear of missing out [48]. Another possible explanation may be related to the fact that individuals addicted to Facebook are likely to have low self-esteem and depression [5, 49]. In this situation, Facebook may be perceived by individuals as a place where they can create their image in the way they would like it to be perceived by others [50]. For this reason, they can use Facebook more and more by using the app on their phone.

We also demonstrated that females are more vulnerable to Facebook addiction. Our results are in line with other studies [5, 27, 28] indicating that female gender is a predictor of Facebook addiction. Moreover, it has been shown that the relationship between time spent on SNSs and Internet addiction is stronger for women than for men [51]. One possible explanation may be that woman, compared to men, spend more time on Facebook than they expected, feel closer to friends on SNSs and feel more dependent on this medium [52]. Also, women perform more ‘liking’, commenting and messaging on Facebook than men [53]. This may lead to the conclusion that women transfer a large part of their social activity to SNSs and thus may be more vulnerable to problematic Facebook use.

We also analysed the relationship between several variables and the time spent using Facebook, but no relation was found. These results may suggest that the variables analysed are more related to Facebook addiction than to the time spent on this SNS. However, further research is needed to provide accurate answers in this area.

In summary, the results of our research showed that impulsivity, the ability to stay focused on self-initiated and pleasant activities, the amount of time spent on Facebook, Facebook smartphone app use and female gender may be predictors of Facebook addiction. In particular, a high impulsivity level in combination with a high ability to stay focused on pleasant activities may contribute to explaining the mechanism leading to addictive Facebook use. These findings may serve as a basis for the design of prevention programmes for individuals at risk of Facebook addiction. Moreover, the results of our research indicate that impulsivity is an important predictor for this type of addiction, which may also be confirmed by other studies on SNS addiction and Internet addiction [10, 43, 44, 54].

Our results should be interpreted in light of several limitations. The data were collected from Polish people and thus the results may not be fully generalizable to Facebook users from other countries. Further research is therefore needed to determine whether similar relationships can be identified in other geographic groups. Also, self-esteem and depression have not been included in the study but it can be assumed on the basis of previous studies that they would also be positively related to Facebook addiction [16, 49]. Moreover, another limitation is associated with the cross-sectional design and self-report measurement. Experimental and clinical studies should therefore be carried out to verify the results obtained in this study.
